# Evaluation of Mouse Oocyte *In Vitro* Maturation Developmental Competency in Dynamic Culture Systems by Design and Construction of A Lab on A Chip Device and Its Comparison with Conventional Culture System 

**DOI:** 10.22074/cellj.2016.4315

**Published:** 2016-05-30

**Authors:** Behnaz Sadeghzadeh Oskouei, Maryam Pashaiasl, Mohammad Hasan Heidari, Mohammad Salehi, Hadi Veladi, Firuz Ghaderi Pakdel, Parviz Shahabi, Marefat Ghaffari Novin

**Affiliations:** 1 Drug Applied Research Center, Tabriz University of Medical Sciences, Tabriz, Iran; 2 Womens Reproductive Health Research Center, Tabriz University of Medical Sciences, Tabriz, Iran; 3 Department of Anatomy and Biology, Shahid Beheshti University of Medical Sciences, Tehran, Iran; 4 Cellular and Molecular Biology Research Centre, Shahid Beheshti University of Medical Sciences, Tehran, Iran; 5 Department of Electronic Engineering, Tabriz University, Tabriz, Iran; 6 Department of Physiology, Urmia University of Medical Sciences, Urmia, Iran; 7 Department of Physiology, Tabriz University of Medical Sciences, Tabriz, Iran

**Keywords:** *In Vitro* Oocyte Maturation, Microfluidics, Lab-On-A-Chip Device, *In Vitro* Fertilization, Glutathione

## Abstract

**Objective:**

In conventional assisted reproductive technology (ART), oocytes are cultured in static microdrops within Petri dishes that contain vast amounts of media. However, the *in vivo* environment is dynamic. This study assesses *in vitro* oocyte maturation through the use of a new microfluidic device. We evaluate oocyte fertilization to the blastocyct stage and their glutathione (GSH) contents in each experimental group.

**Materials and Methods:**

In this experimental study, we established a dynamic culture condition. Immature oocytes were harvested from ovaries of Naval Medical Research Institute (NMRI) mice. Oocytes were randomly placed in static (passive) and dynamic (active) *in vitro* maturation (IVM) culture medium for 24 hours. *In vitro* matured oocytes underwent fertilization, after which we placed the pronucleus (PN) stage embryos in microdrops and followed their developmental stages to blastocyst formation after 3 days. GSH content of the *in vitro* matured oocytes was assessed by monochlorobimane (MCB) staining.

**Results:**

We observed significantly higher percentages of mature metaphase II oocytes (MII) in the passive and active dynamic culture systems (DCS) compared to the static group (P<0.01). There were significantly less mean numbers of germinal vesicle (GV) and degenerated oocytes in the passive and active dynamic groups compared to the static group (P<0.01). Fertilization and blastocyst formation rate in the dynamic systems were statistically significant compared to the static cultures (P<0.01). There was significantly higher GSH content in dynamically matured oocytes compared to statically matured oocytes (P<0.01).

**Conclusion:**

Dynamic culture for *in vitro* oocyte maturation improves their developmental competency in comparison with static culture conditions.

## Introduction

Assisted reproductive technology (ART), through direct manipulation of gametes/embryos outside the body with the intent to establish pregnancy (1), plays a critical role during *in vitro* production of domestic animals and human infertility treatments This technique has considerable advantages for individuals incapable of having children via the natural reproductive cycle. In the process, infertile couples go through profound periods of distress (2). ART is expensive and, unfortunately, has a relatively low success rate, particularly for humans (3). Thus far, all efforts and advancements in this area focus on modifications in media ingredients while there have not been considerable changes in the physical instruments used in handling and manipulation of oocytes/embryos in ART laboratories (4,5). Unfortunately, these laboratories still use polystyrene test tubes, Petridishes, and fine-boreglass-pipettes as static culture systems (3). 

Obviously, the dynamic environment of tubes/ uterus produces a unique condition capable of supporting embryo development. This environment can also modulate gene expression (6) and interrupt cell-surface gradients which encompass embryos (2). These gradients such as potassium, calcium and oxygen also exist in traditional static culture conditions, possibly through secretion of trophic autocrine/paracrine factors; however, because of the nature of static conditions, the above mentioned gradients cannot be disturbed, and consequently cannot provide a more homogenous environment similar to *in vivo* dynamic culture systems (DCSs). The *in vitro* culture is maintained at higher O_2_ concentrations than the *in vivo* environment and results in increased production of reactive oxygen species (ROS). During fertilization, glutathione (GSH) content in oocytes participates in sperm decondensation parallel to oocyte activation. It is the major nonprotein sulfhydryl compound in mammalian cells and has a well-known role in protecting the cells from ROS damage (1). Therefore, natural movement results in mechanical effects such as shear stress (SS), agitation and friction in the uterine tube which usefully decrease oxidative stress and improve the development of culturing cells (2). Even though *in vivo* embryo development occurs in a dynamic environment, oocyte/embryo culture in traditional static conditions is feasible (6,7). However, studies with animal models have indicated that *in vitro* grown oocytes/embryos are less efficient compared to *in vivo* oocytes/embryos (6). Accordingly, it seems that static oocytes/embryos culturing systems have to change so as to better mimic the naturally dynamic environment of the mammalian female reproductive tract (8). There are important differences between static (conventional) and dynamic (*in vivo* environment) culture conditions (2). *In vitro* culture of mammalian oocytes/embryos aretypically performed in stagnant conditions under a layer of mineral oil, with large volumes of the medium which increase ROS compared to the sub-microliter amounts or restricted moist environment of *in vivo* conditions that occur within the lumen of the female reproductive tract (5,9,10). Oil is an essential component for static gametes and the embryo culture. It considerably limits medium evaporation and contributes to cell monitoring due to its transparency. However, there are lipophilic factors in the medium that have the ability to become absorbed into the oil overall. Conversely, hazardous factors in the oil exist that have the capability of diffusing into the medium (10,11). In such a static system, numerous physical limitations such as oil and medium mixing, high medium volume, low surface area-to-volume (SA/V) ratios (10,12), and inert conditions which can potentially increase to develop “bedsores”, a doubted but yet unproven explanation for low success rates in ART (3,5,13). What can address this issue is the development of a modern microfluidics technology by device miniaturization or the so-called lab-on-a-chip (LOC) and the creation of a dynamic environment (2,14). Microand nanofluidics have substantially improved over the last decades and are successful in their research-oriented scope along numerous dimensions such as drug delivery, genetic analysis, sperm sorting, and animal *in vitro* fertilization (IVF). Various efforts have been made to build devices useful for oocyte maturation and embryo culture (2,6,15). Nonetheless, dynamic microfluidic devices are not yet available on a mass scale for use in clinical embryology laboratories (15). Recourse to this modern technology and related devices in ART to improve outcomes began almost a decade ago (6). Collectively, LOC technology is a promising approach since the devices appear to contribute to oocyte/ embryo development in amazing and unique ways. They are probably innovative sources for embryology laboratories (8,12). LOC systems can mimic oviduct functions and structures (2). 

Oocyte maturation is the most important stage because it determines subsequent successful fertilization, zygote formation, and suitable transition to the blastocyst stage, as well as appropriate implantation (6,16). Many factors influence the fertilization ability of oocytes. The aim of this study is to evaluate the effects of a microfluidic culture system on oocyte *in vitro* maturation (IVM), preimplantation embryo development to the blastocyst stage, and its comparison to a conventional static system. We have measured GSH content in the *in vitro* matured oocytes. 

## Materials and Methods

### Animals

In this experimental research, we obtained oocytes from 6 to 8 week-old Naval Medical Research Institute (NMRI) mice (Pasteur Institute, Iran). Sperm were acquired from males, 8-10 weeks of age. Animals were kept under controlled 12-hour light/dark conditions at constant temperature and relative humidity with free access to water and food. All experiments were performed in strict accordance with the Ethical Committee and guiding principles for the care and use of research animals adopted by the Shahid Beheshti University of Medical Sciences (16) Tehran, Iran. 

### Superovulation, oocyte collection, in vitro maturation, and in vitro fertilization

In order to retrieve immature oocytes, we subjected the mice to induced superovulation. Mice received intraperitoneal (i.p.) injections of 10 IU pregnant mare’s serum gonadotropin (PMSG, Gonaser^®^, Laboratorios Girona, Spain). Oocytes were retrieved 45-48 hours after PMSG injection. 

Animals were killed by cervical dislocation. Their ovaries were dissected out and placed in tissue culture dishes (BD Falcon, 35×10 mm) that contained human tubal fluid (HTF) 4-(2-Hydroxyethyl)-1-piperazineethanesulfonic acid (HEPES) buffered (GC-HTF W/HEPES, Genocell Ideal, Iran) supplemented with 10% (v/v) qualified fetal bovine serum (FBS, Gibco, Invitrogen, South America). Cumulus oocyte complexes (COCs) were released by follicular puncturing with the aid of a pair of 28 G needles as visualized under a stereomicroscope (Olympus, Japan) (17). We chose only cumulus intact oocytes in the GV stage that had evenly granulated cytoplasm. These oocytes were transferred to maturation medium (16). After several washes, we selected only fully expanded COCs (17,19) which were divided randomly and synchronously into dynamic (passive and active) and static culture system groups. In the dynamic groups, we placed the COCs in microfluidic chips that contained 15-20 µL of HTF medium (GC-HTF, GenocellIdeal, Iran) supplemented with 10% (v/v) qualified FBS, 10 µg/mL follicle stimulating hormone (FSH), 10 µg/mL luteinizing hormone (LH), and 1 µg/mL estradiol-17ß (Sigma, St. Louis, MO, USA) as IVM medium (5 oocytes/15 µL medium). COCs were incubated in a humidified atmosphere with 5% CO_2_ at 37˚C for 20-24 hours (20). In the active dynamic group, we used a low flow rate peristaltic pump (Langer Instruments, USA) which produced a pulsatile fluid movement (1 µL/hour). However, in the passive dynamic group, we did not use any pump. In this group, the fluid movement was created by gravity. In the static group, COCs were placed in several droplets of IVM medium (15 oocytes/50 µL medium under mineral oil) (16,19) in tissue culture dishes, which were incubated under conditions similar to the experimental (dynamic) groups for 20-24 hours. We assessed maturation status in the three groups according to polar body extrusion by phase contrast inverted microscope (Euromex, Netherlands). 

IVF was performed as follows. The metaphase II (MII) oocytes obtained from dynamic and static groups were fertilized by sperm acquired from the cauda epididymides of 8-10 week-old mice. *In vitro* matured oocytes were transferred to T6 medium (Royan Institute, Iran) supplemented with 15% bovine serum albumin (BSA, Sigma, St. Louis, MO, USA), after which they were placed in microdrops which contained 15% T6. Sperm were prepared in 15% T6 medium by the swim up method and then added to the microdrops. After 5-6 hours, pronucleus (PN) stage embryos were randomly transferred to the different groups that contained T6 medium supplemented with 4% BSA as the IVF medium, then cultured for 72 hours. 

### Assay for glutathione content 

We estimated the GSH concentration in oocytes by using a fluorescent indicator of GSH, monochlorobimane (MCB). *In vitro* matured oocytes obtained from three groups were incubated with 50 mM MCB in flushing holding medium (FHM) for 45 minutes. We subsequently recorded MCB fluorescence at 390 nm by a digital camera. Fluorescence intensity was analyzed by Image J software (National Institutes of Health, Bethesda, MD, USA) (16). 

### Device design, fabrication and fluid actuation

The device was fabricated by standard soft lithography procedures. In order to design the microfluidic system we used a microchannel as conduits for fluid flow through a microfunnel where COCs reside. Poly (dimethylsiloxane) (PDMS, Sylgard 184, Dow Corning, USA) (21) was chosen for this microfluidics system microfabrication because of its favorable mechanical properties, optical transparency, biocompatibility (22,23), and straightforward manufacturing by rapid prototyping (24). PDMS is particularly appropriate for oxygenator application because of its high permeability to oxygen gas (25). Liquid PDMS prepolymer solution was poured on to the master, degassed in a vacuum pump, and cured at 70˚C for 1.5 hours, then peeled off the master. The upper and lower layer surfaces were treated in a corona generator and bonded together. Two medium reservoirs were fixed to the inlet and outlet on the upper layer. We selected the present culture microfunnel design rather than culture channels in order to minimize excessive fluid mechanical stress that might be associated with passage through narrow channels in previous studies (7). Relief features of desired channel structures were composed of SU-8 (MicroChem, Newton, MA, USA) and fabricated on a thin glass wafer. In order to construct the two-layer microfluidic device, a 10:1 monomer/ hardener mixture was mixed and degassed, then applied via spin-coating at a thickness of 120 mm onto an SU-8 mold that contained the fluid channels. Simultaneously, a 5:1 PDMS mixture was prepared and poured to a thickness of ~ 6 mm onto a second SU-8 mold that contained the gas channels (26). 

**Fig.1:**
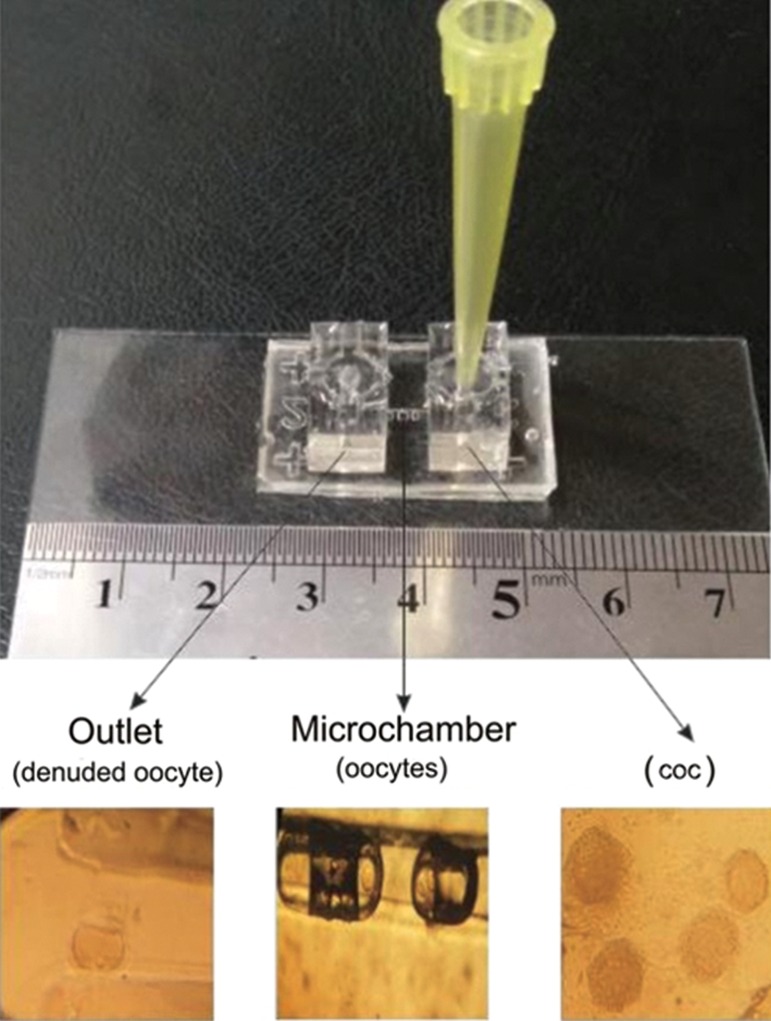
Different sections of the microfluidic device. The inlet port included cumulus oocyte complexes (COCs). The microchamber included oocytes at the germinal vesicle (GV) stage. The outlet port included denuded oocytes.

Two types of devices (passive and active) consisted of two PDMS layers with a microchannel in the upper layer and a microchamber array in the lower layer. The microchannel was 7 mm in length, 140 μm in width, and 200 μm in depth to transport the oocytes. The inlet and outlet were connected with the microchannel (Fig.1). The square-shaped microchambers were 120 μm in width and various lengths (120, 360, 600 and 900 μm), at a 250 μm interval to trap the oocytes. Although the four microchambers were designed to compare their performance in oocyte entrapment and developmental competency of COCs, in groups 1, 3, 5 and 7, the results were not significant (data not shown). 

During the experiment, we loaded the oocytes into the funnel shaped inlet port, after which they were transported through the microchannel and subsequently lodged in the microchamber array (Fig.1). We used a 100 µL tipfor the medium reservoir. 

### Computational model 

We constructed the finite element models (COMSOL 3.5, Comsol AB, Burlington, MA, USA) and used 2D simulations to study the flow behavior in the biochip with different depths. 

The model was based on the steady-state Navier–Stokes equation for an incompressible Newtonian fluid (25): 

ρ(υ.s)υ=-sp+ƞs2υ

Where υ is the velocity vector field, p is the pressure, ρ is the medium density, and ƞ is the dynamic viscosity. 

In addition, computational fluid dynamics was used to predict the wall SS as a function of the channel width and the flow rate. Since the channel length (7 mm) in the device was significantly greater than the channel height (200 μm) the system could be successfully modeled using 2D simulation. Under these conditions, we used this equation to estimate the maximum SS applied to the cell (τs) (25): 

$$\mathrm{\tau s}=\frac{\left(6\times 2.95\mathrm{\mu Q}\right)}{H^{2}}$$

Where H is the channel height, μ is the medium viscosity and Q is the flow rate. 

For an inlet velocity of 0.5 mm/seconds, the maximum wall SS inside the microchannel was 0.04 dynes/cm^2^, which was considerably lower than the average wall SS amplitude that an oocyte could endure (dynes/cm^2^) (25). 

### Statistical analysis

All statistical analyses were performed using SPSS 22 for Windows (Chicago, IL, USA). IVM, fertilization, blastocyst formation rate and GSH levels in the static and dynamic groups were compared by ANOVA followed by the Tukey post hoc test. Data were normalized [(oocyte number/stage/ total number of oocytes in the Petridish or microchip)* 100]. Data are expressed as means ± SE. A statistically significant difference was accepted at P<0.05. 

## Results

### Oocyte maturation

**Fig.2:**
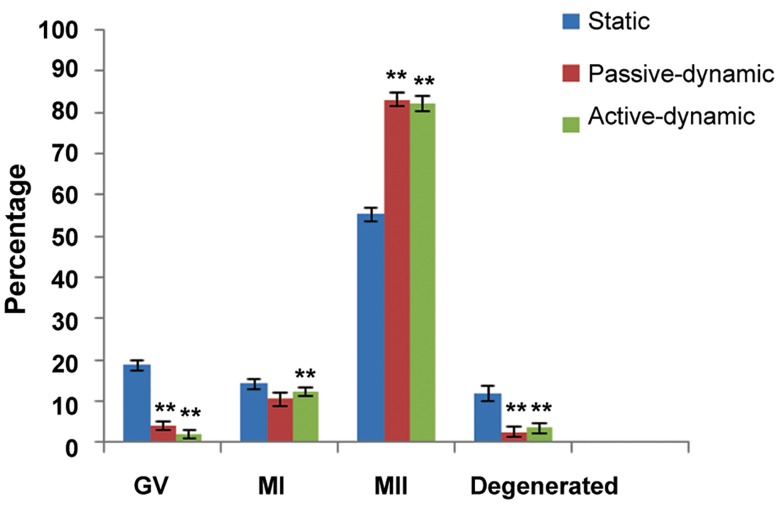
Percentage of different stages of oocytes in the three groups. **; P<0.01 (passive dynamic and active dynamic vs. static system), MI; Metaphase I, MII; Metaphase II and GV; Germinal vesicle.

There were 955 total COCs retrieved from mice ovaries. We transferred 495 COCs to the static IVM medium and 460 COCs to the dynamic (230 COCs to active and 230 COCs to passive) IVM medium synchronously. After 20-24 hours of maturation, we observed a significantly higher percentage of MII oocytes in the passive (83.04%) and active (82/17%) dynamic groups compared to the static system (55.35%). However, there were significantly lower (P<0.01) results for the percentage of oocytes that arrested in the germinal vesicle (GV) stage for the passive (3.91%) and active (2.17%) dynamic groups compared to the static system (18.78%), as well as degenerated oocytes in the passive (2.6%) and active (3.47%) dynamic groups compared to the static system (11.91%).We observed no significant difference in GV breakdown (GVBD) oocytes between the passive (10.43%) and active (11.3%) dynamic groups compared to the static system (14.34%, Fig.2). Interestingly, our results showed that COCs during their movement through the microchannel lost their cumulus cells and were denuded when they arrived at the outlet (Fig.1), therefore we did not need hyaluronidase enzyme. 

### In vitro fertilization

**Fig.3:**
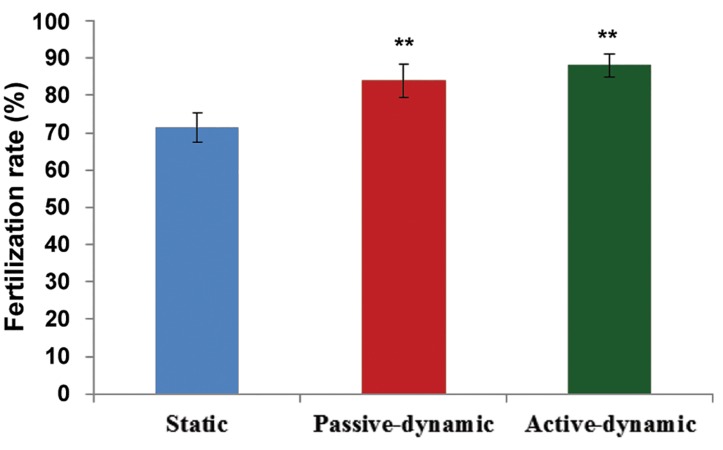
Fertilization rate (%) in the three groups. **; P<0.01 (passive-dynamic and active-dynamic vs. the static system).

**Fig.4:**
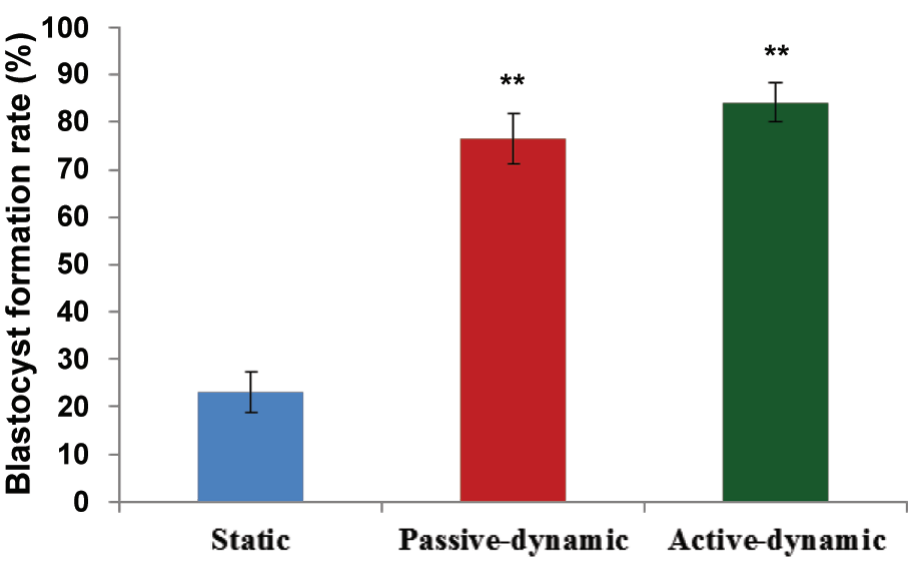
Blastocyst formation rate (%) in the three groups. **; P<0.01 (passive-dynamic and active-dynamic vs. static system).

A total of 135 staticand187 dynamic mature MII oocytes were fertilized; the PN stages oocytes were randomly and synchronously transferred to different groups. There were significantly higher fertilization rates in the passive dynamic group (84%) and active dynamic group (88%) compared to the static group (71.36%, P<0.05). However, this difference between the two dynamic groups was not significant (P<0.05, Fig.3). Our findings showed significantly higher blastocyst formation rate in the passive dynamic group (76.58%) and active dynamic group (84%) compared to the (P<0.05) static group (22.92%, Fig.4). 

### Glutathione levels in the oocytes

**Fig.5:**
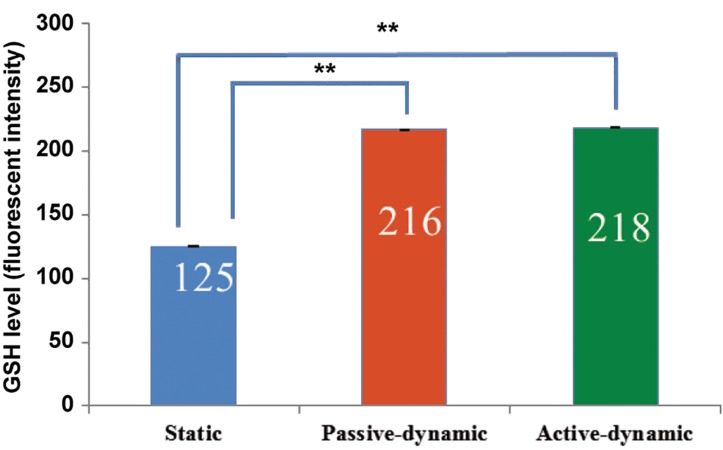
Glutathione (GSH) level (average pixel intensity) of *in vitro* matured oocytes in active dynamic, passive dynamic and static groups. The GSH level was evaluated by monochlorobimane (MCB) staining. **; P<0.01 (passive-dynamic and active-dynamic vs. static system).

**Fig.6:**
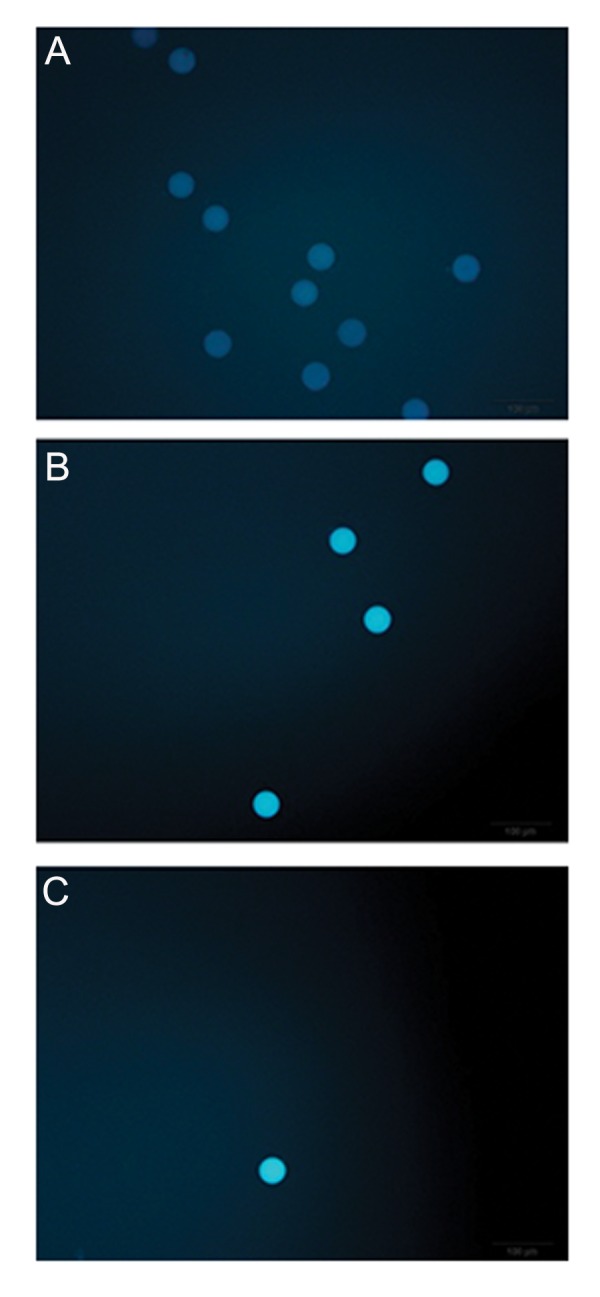
Fluorescent intensity of monochlorobimane (MCB) stained mature oocytes. A. Static, B. Passive-dynamic, and C. Active-dynamic.

The dynamic culture condition had a significant (P<0.05) effect on the level of GSH in the *in vitro* matured oocytes (Fig.5,6). 

## Discussion

Due to benefits of microfluidic devices and *in vitro* oocyte maturation prominence, we constructed a new device for IVM. 

It has been reported that a dynamic culture of pre-implantation embryos has a role in successful implantation and ongoing normal pregnancies because of the acquisition of developmental competence. However, few studies focused on the dynamic culture of immature oocytes (27). The present findings showed that the dynamic culture during IVM efficiently improved oocyte development. 

Embryos obtained from dynamic matured oocytes have shown higher development to the blastocyst stage which agreed with previous studies (28,29). To the best of our knowledge, this study is the first that has directly shown the involvement of the dynamic culture of immature oocytes in protection of mice oocytes against cell damage caused by ROS during IVM. Our results showed significantly increased GSH content in oocytes after 24 hours of maturation. As previously reported, the generation of ROS during *in vitro* culture was thought to cause reduced sperm motility, lipid peroxidation, decreased capacity of sperm-oocyte fusion, and retarded embryo development (30). 

Oocyte maturation is the most important stage because it determines subsequent successful fertilization, zygote formation, and suitable transition to the blastocyst stage, as well as appropriate implantation (6,16). Oocyte maturation is a wellregulated event that includes nuclear and cytoplasmic maturation (20). Occasionally, infertility occurs due to lower oocyte capability in terms of maturation. Within the female reproductive tract, the ovulated oocyte/preimplantation embryo is unique because it is free-floating, lacks a blood supply, continuously moves through a changing fluid environment (27,28) and develops in the lack of straight cell communication with fallopian tube epithelial cells (7). Thus far, there have been encouraging consequences mostly with microfluidic pre-implantation embryo culture technology and also constructed devices were somewhat complicate (29). Accordingly, application of the lab on a chip (LOC) system in reproductive biology provides new possibilities for the development of techniques to assess the developmental competency of mammalian oocytes. This system may provide controllable microenvironments specialized for embryo development in addition to an automated platform for performing the multiple IVF steps (31,34). 

Willadsen (34) first reported on the importance of the microenvironment and embryo handling/culture in the 1970s. They reported that agar coating of embryos improved embryo development (7,34). Choi et al. (35) developed a microfluidic device capable of selecting normal oocytes with relatively high specificity. Similarly, intrinsic sperm mobility and microfluidic laminar flow were used to isolate motile sperm from non-motile sperm, debris and seminal plasma (14,36). Zeringue et al. (37) developed a microfluidic platform for control of embryo positioning, movement, and zona pellucida removal for chimeric and transgenic production. Although these devices provide convenient handling properties for sperm, oocytes and embryos, they did not address the potential of microfluidics technology to their developmental competency. Collectively, results from this comparative controlled research suggested that the microenvironment obtained by microfluidics supports enhanced immature oocyte development compared to conventional static culture conditions. The greatest development of immature oocytes has been shown to occur in small volumes or in the presence of multiple similar cells, which is likely due to the beneficial effects of autocrine factors (38,39). Known autocrine factors include leukemia inhibitory factor (40), interleukin-1, insulin-like growth factor (41), platelet derived growth factor (42), epithelial growth factor (EGF) (43), and transforming growth factor (44). Our findings have also confirmed those of previous studies. We demonstrated that fluid movement and mechanical agitation of immature oocytes during dynamic culture could improve their development. We observed significantly lower mean GV values and degenerated oocytes in passive and active dynamic groups compared to the static group (P<0.01). However, none of the variations between passive and active dynamic groups were significant. This result could be due to controlled fluid movement. This seemed to be the result of the relative equal velocity in the two types of chips (1 µL/minute) (6). Therefore, passive dynamic culture appeared better than the active system due to its simplicity and cost effectiveness. 

Mainly, fluid movement and mechanical agitation of reproductive cells during the culture can disrupt concentration gradients of substrates, secretory molecules, and dissolved gases. Movement may ensure that unstirred layers do not form around the embryo and possibly facilitate exchange of gases and/or metabolites (6). Therefore, it seems that the use of DCS in ART laboratories is of benefit, particularly for patients suffering from oocyte maturation deficiency such as those diagnosed with polycystic ovary syndrome. 

We propose research of embryo transfers to miceuteri with subsequent assessment of the pregnancy rate in the various study groups. 

## Conclusion

We have fabricated a new, easy-to-use microfluidic device for an immature oocyte culture. Its simple architecture, chip design, and programmability of fluid actuation of the present dynamic system, in particular the passive type, allows for suitable and feasible manipulation of chemical and mechanical microenvironments for *in vitro* oocyte maturation. The present study has shown that dynamic culture for *in vitro* oocyte maturation increases MII obtained oocytes in comparison with the conventional static culture system. This system increases fertilization, blastocyst formation rate, and GSH content of *in vitro* matured oocytes. The passive dynamic culture system because of its simplicity appears to be better than the active system. 
